# Clinical use of the VNtyper-Kestrel pipeline for *MUC1* variant detection in autosomal-dominant tubulointerstitial kidney disease

**DOI:** 10.1007/s10157-025-02675-y

**Published:** 2025-04-17

**Authors:** China Nagano, Naoya Morisada, Yuta Inoki, Yu Tanaka, Yuta Ichikawa, Chika Ueda, Hideaki Kitakado, Yuya Aoto, Nana Sakakibara, Tomoko Horinouchi, Tomohiko Yamamura, Shingo Ishimori, Kandai Nozu

**Affiliations:** 1 Department of Pediatrics, Kobe University Graduate School of Medicine, 7-5-2 Kusunoki-Cho, Chuo-Ku, Kobe, Hyogo 650-0017 Japan; 2 Department of Clinical Genetics, Hyogo Prefectural Kobe Children’s Hospital, 1-6-7 Minatojima-Minamimachi, Chuo-Ku, Kobe, Hyogo 650-0047 Japan

**Keywords:** ADTKD, MUC1, VNtyper, Variable number tandem repeat

## Abstract

**Background:**

Autosomal-dominant tubulointerstitial kidney disease caused by *MUC1* (ADTKD-*MUC1*) is a rare disorder characterized by progressive kidney dysfunction. Pathogenic variants in *MUC1* are difficult to detect owing to the variable number tandem repeat region. To address this issue, VNtyper-Kestrel, a bioinformatics pipeline for short-read sequencing data, was recently developed. In this study, the performance of VNtyper-Kestrel for detecting *MUC1* variants in clinical settings was evaluated.

**Methods:**

We used VNtyper-Kestrel to retrospectively analyze short-read sequencing data for 209 individuals with suspected ADTKD who were previously evaluated through long-read sequencing. Data from a panel including ~ 180 genes and an ADTKD-specific panel were used. In addition, the pipeline was applied to 976 patients with suspected hereditary kidney diseases other than ADTKD and positive cases were validated using long-read sequencing. Accuracy was assessed by comparisons with the results of long-read sequencing.

**Results:**

Using VNtyper-Kestrel, we identified *MUC1* variants in 16 of 19 confirmed cases of ADTKD-*MUC1*. Three initially negative cases were reanalyzed using the ADTKD-specific panel, yielding positive detection results with high confidence. We obtained two low-confidence positive results from 190 cases of suspected ADTKD and 10 low-confidence positive results among 976 non-ADTKD cases; however, all were classified as false positives upon long-read sequencing validation.

**Conclusions:**

VNtyper-Kestrel demonstrated high sensitivity in identifying *MUC1* variants when sequencing coverage was adequate, supporting its potential as a rapid and cost-effective screening tool. However, confirmatory long-read sequencing is needed in uncertain cases. Optimizing coverage and refining patient selection criteria could improve the clinical utility of this approach.

**Supplementary Information:**

The online version contains supplementary material available at 10.1007/s10157-025-02675-y.

## Introduction

Autosomal-dominant tubulointerstitial kidney disease (ADTKD) is a rare autosomal-dominant kidney disease with largely non-specific clinical, laboratory, and histological findings [1, 2]. A gene-based subclassification was established by KDIGO in 2015 and diagnostic criteria were proposed [1]. *UMOD*, encoding uromodulin [3]; *MUC1*, encoding the glycoprotein mucin 1 [4]; *HNF1B*, encoding the transcription factor hepatocyte nuclear factor 1β [5]; *REN*, encoding preprorenin, the precursor of renin [6]; and *SEC61A1*, encoding the alpha1 subunit of the SEC61 complex [7] have been identified as causative genes for ADTKD.

Previous studies have identified ADTKD-*MUC1* and ADTKD-*UMOD* as the most common subtypes of ADTKD [8]. ADTKD-*MUC1* (OMIM:174,000) is caused by a pathogenic variant in the *MUC1* gene. Its prevalence is unclear because variants in *MUC1* are difficult to detect using next-generation sequencing as well as conventional Sanger sequencing, and require specialized genetic testing [4]. Based on a cohort of 95 patients from 24 families, ADTKD-*MUC1* shows an age of onset of end-stage kidney disease (ESKD) ranging from 16 to 80 years and a 24% prevalence of gout [9]. In a Spanish cohort of 90 patients with ADTKD-*MUC1* (16 families), the median renal survival age was 51 years [8]. A study in Europe and the USA involving 80 patients with ADTKD-*MUC1* showed a median renal survival age of 46 years [10]. In Japan, a cohort of 20 patients with ADTKD-MUC1 showed a median renal survival age of 47 years [11].

*MUC1* encodes mucin 1, a heavily glycosylated transmembrane protein that forms a protective mucous barrier on the apical side of epithelial cells and may be involved in intracellular signaling, cell–cell interactions, modulation of immune functions, and sensing of the extracellular environment. In the human kidney, mucin 1 is expressed from the thick ascending link to the collecting ducts [12]. *MUC1* consists of 60 base pair GC-rich variable number tandem repeats (VNTRs), encoding an extracellular domain rich in threonines and/or serines with heavy *O*-glycosylation [13]. The number of 60 base pair repeats is highly variable, ranging from 20 to 125 [4]. Most pathological variants are caused by a single nucleotide C insertion in the VNTR region of *MUC1*, resulting in a frameshift and a truncated protein (MUC1-fs) that accumulates in intracellular vesicles and causes tubulointerstitial damage [14].

Finding pathogenic variants in VNTRs using standard Sanger sequencing or massively parallel short-read sequencing is difficult because of the characteristics of VNTRs, including their high guanosine/cytosine content. These variants can be detected using a mass spectrometry-based assay developed by Blumenstiel et al. [15]; however, due to its technical specificity, this method can only be implemented in a few centers around the world. Recent reports have indicated that long-read sequencing can effectively detect *MUC1* variants [13, 16, 17]. Alternatively, pathogenic *MUC1* variants can be detected rapidly from short-read sequencing data using VNtyper-Kestrel, proprietary bioinformatics software [18].

Our aim was to evaluate the effectiveness of short-read sequencing and VNtyper-Kestrel for detecting pathological variants in ADTKD-*MUC1*. This approach provides a basis for addressing the large number of undiagnosed cases of ADTKD-*MUC1* linked to the limitations of VNTR analyses.

## Materials and methods

### Study participants

After obtaining informed consent, clinical data and blood samples were collected from individuals with suspected hereditary kidney diseases in Japan. The study was approved by the Institutional Review Board of Kobe University Graduate School of Medicine (IRB approval number 301). Patients were enrolled between January 2015 and October 2024.

**Fig. 1:**
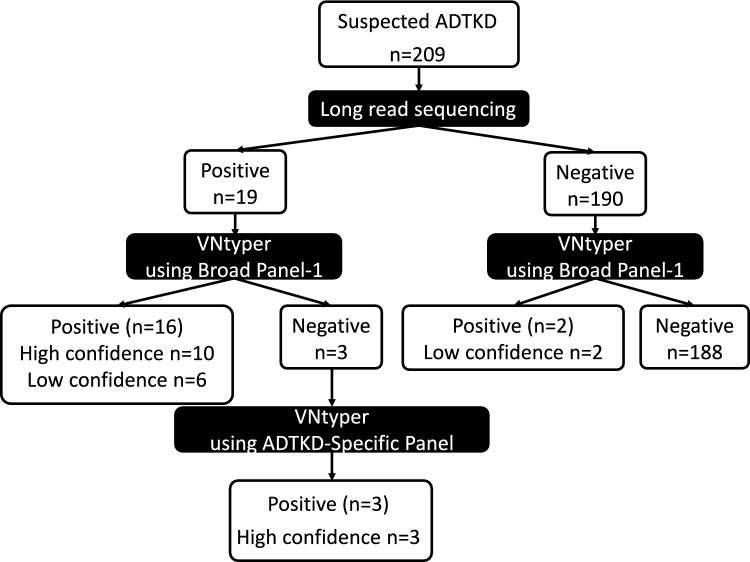
First strategy for assessing the diagnostic performance of the VNtyper-Kestrel pipeline. We retrospectively analyzed short-read sequencing data for patients with suspected autosomal-dominant tubulointerstitial kidney disease (ADTKD) who had previously undergone long-read sequencing. These cases were screened using broad panel-1. In cases where broad panel-1 yielded negative results, an ADTKD-specific panel was applied, followed by reanalysis using VNtyper-Kestrel

The performance of the VNtyper-Kestrel pipeline was validated using two approaches with distinct clinical cohorts. In the first validation approach (Fig. 1), we retrospectively analyzed short-read sequencing data using a broad panel (referred to as broad panel-1) (Supplementary Table S1) for individuals with suspected ADTKD with no mutations detected in ADTKD genes other than *MUC1.* The patients had previously undergone long-read sequencing for diagnostic purposes (n = 209). These patients were selected based on clinical characteristics consistent with ADTKD, including a family history of autosomal-dominant kidney disease, chronic kidney disease (CKD) with tubulointerstitial features, and the absence of significant glomerular abnormalities. In total, 19 individuals harbored *MUC1* VNTR region variants, as detected by long-read sequencing. Short-read sequencing data for these individuals were reanalyzed using VNtyper-Kestrel to assess its accuracy in detecting *MUC1* variants. In cases that tested negative using broad panel-1, short-read sequencing was performed using an ADTKD-specific gene panel (Supplementary Table S2) followed by analyses using VNtyper-Kestrel to improve variant detection.

**Fig. 2:**
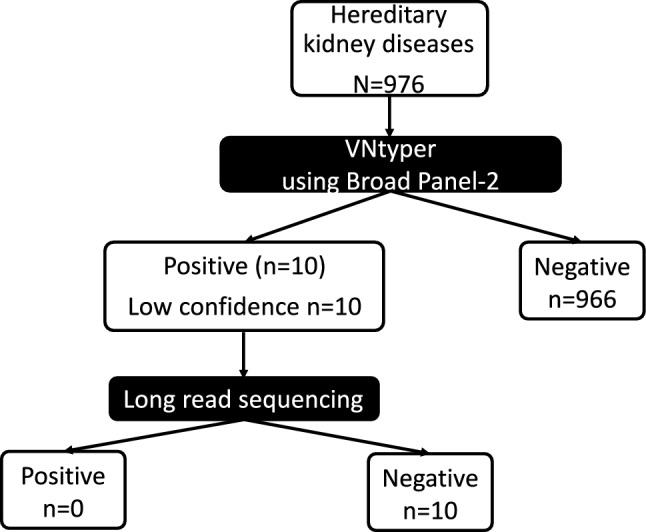
Second strategy for assessing the diagnostic performance of the VNtyper-Kestrel pipeline. We applied VNtyper-Kestrel to short-read sequencing data for patients suspected of having hereditary kidney diseases other than ADTKD, using broad panel-2 for initial screening. Cases identified as positive using VNtyper-Kestrel underwent long-read sequencing for validation

In the second validation approach (Fig. 2), we applied VNtyper-Kestrel to short-read sequencing data using another large gene panel (broad panel-2) (Supplementary Table S3) for individuals with suspected hereditary kidney diseases other than ADTKD who had undergone genetic testing (*n* = 976). In cases where VNtyper-Kestrel identified *MUC1* variants, long-read sequencing was performed to validate whether these represented true-positive cases.

This dual validation strategy enabled a comprehensive assessment of the reliability and diagnostic accuracy of VNtyper-Kestrel across different clinical contexts, reinforcing its potential utility in the genetic diagnosis of hereditary kidney diseases.

## Genetic analysis

### Short-read sequencing

Genomic DNA was extracted from peripheral blood using the Quick Gene Mini 80 system (Wako Pure Chemical Industries, Ltd., Tokyo, Japan) and used for targeted sequencing and Sanger sequencing. Targeted sequencing using next-generation sequencing (NGS) was conducted for genes responsible for inherited kidney diseases. The sample library for NGS was prepared using HaloPlex or SureSelect custom capture library 0.5–2.9 Mb (Agilent Technologies, Santa Clara, CA, USA), in accordance with the manufacturer’s workflow. All indexed DNA samples were amplified by polymerase chain reaction and sequenced using the MiSeq platform (Illumina, San Diego, CA, USA).

### Long-read sequencing of the *MUC1* VNTR region

Long-read sequencing of the *MUC1* VNTR region was performed using the PacBio Sequel II System or Revio System (Pacific Biosciences, Menlo Park, CA, USA). Primers were designed according to a previous study [17].

For library preparation, sequencing adapters were ligated to both ends of the amplicons using PacBio Sequel II Sequencing Kit 2.0 or SMRTbell® Prep Kit 3.0. The sequencing templates were prepared by binding the polymerase to the adapters, and sequencing was performed on an SMRT cell using the PacBio Sequel II System or Revio System. Library preparation, sequencing, and bioinformatics analyses were performed by Takara Bio (Kusatsu, Shiga, Japan).

The nucleotide sequences, read counts, and allele frequencies for each patient were compiled into an Excel file. Each identified sequence was manually reconstructed at 60 bases per line, as described by Kirby et al. [4].

### VNtyper-Kestrel pipeline

The VNtyper-Kestrel pipeline, a bioinformatics tool designed to detect pathogenic variants in the *MUC1* gene, was used to evaluate short-read sequencing data. This approach is particularly advantageous for analyzing the GC-rich VNTR region of *MUC1*, which poses challenges for conventional sequencing methods. The VNtyper-Kestrel pipeline uses a k-mer-based strategy to identify unique sequence signatures associated with pathogenic variants, specifically detecting aberrant nucleotide insertions causing characteristic *MUC1* frameshift variants. The pipeline reconstructs the VNTR structure from short-read data and assigns confidence scores to facilitate the prioritization of candidate pathogenic variants. This methodology was previously described by Saei et al. in 2023 [18]. In the Kestrel algorithm, a variant is classified as High Confidence if it meets the following criteria: depth score of at least 0.00515 and estimated depth of the alternate variant greater than 20. All variants that do not meet these criteria are classified as Low Confidence.

In this study, the VNtyper-Kestrel pipeline was applied to short-read sequencing data for patients with suspected hereditary kidney diseases. The variants were then validated using long-read sequencing to assess the accuracy and reliability of the pipeline in a clinical diagnostic setting.

## Results

### First approach (Fig. 1, Supplementary Tables S4, S5)

Among the cases subjected to short-read sequencing, long-read sequencing data were available for 209 cases. Pathogenic variants in *MUC1* were previously identified in 19 of these cases by long-read sequencing. Using broad panel-1 data and VNtyper-Kestrel, 16 of the 19 confirmed cases of ADTKD-*MUC1* tested positive, whereas 3 cases tested negative. The median coverage of the VNTR region of *MUC1* was 344 using broad panel-1. In a re-analysis using the specific panel and VNtyper-Kestrel, all three negative cases were found to be positive. The median coverage of the *MUC1* VNTR region obtained using the ADTKD-specific panel was 2,424.

Among the 190 cases in which no pathogenic *MUC1* variant was identified by long-read sequencing, two cases were VNtyper-positive. However, these results were categorized as low-confidence positives, suggesting they were false positives.

### Clinical characteristics of diagnosed cases (Table 1)

**Table 1:** Clinical characteristics of diagnosed case

No	ID	Age at diagnosis	Sex	Family history of CKD	Pathologic diagnosis/findings	Hypertension Age at diagnosis	Hyperuricemia Age at diagnosis	eGFR	UP	Serum potassium
1	SC370	48	M	+	–	–	ND	RRT (32 y)	0.03	4.1
2	SC416	33	M	+	Chronic TIN	–	+ (29 y)	23	1.6	4.6
3	SC489	54	M	+	Chronic damage of interstitium	+ (54 y)	+ (53 y)	6.5	1.4	5.6
4	SC534	41	F	+	MCKD	+ (40 y)	–	RRT (40 y)	0.27	3.9
5	SC656	46	F	+	–	+ (40 y)	+ (ND)	41	0.12	4
6	SC512	37	M	-	MCKD	–	+ (ND)	16.4	0.31	4.8
7	SC566	50	M	-	MCKD	+ (42 y)	–	10	0.27	3.8
8	SC265	43	F	+	Global sclerosis	–	–	34.8	negative	5.4
9	SC359	39	M	+	MCKD	+ (36 y)	+ (34 y)	40	1.6	4.1
10	SC696	48	F	+	–	+	–	9.9	0.05	5.1
11	SC732	68	F	+	–	+ (50 y)	–	RRT (65 y)	ND	4
12	SC798	48	F	+	MGA	ND	ND	RRT (48 y)	1.38	4.48
13	SC870	25	M	+	–	+ (23 y)	ND	24.5	0	4.8
14	SC874	40	M	+	–	–	–	RRT (31 y)	Auria	4.2
15	SC912	39	F	+	MCKD	–	–	22	Negative	4.8
16	SC973	26	F	+	MCKD	+ (26 y)	–	49.2	0.1	4.3
17	SC995	46	M	+	–	+ (around 20 y)	+ (around 20 y)	RRT (46 y)	0.19	4.4
18	SC1079	26	M	+	MCKD	–	–	47	0.05	3.9
19	SC1112	31	M	+	MCKD	–	–	RRT (28 y)	1.52	4.9

*CKD* chronic kidney disease, *MCKD* medullary cystic kidney disease, *MGA* minor glomerular abnormality, *ND* no data, *RRT* renal replacement therapy, *TIN* tubulointerstitial nephritis

**Fig. 3:**
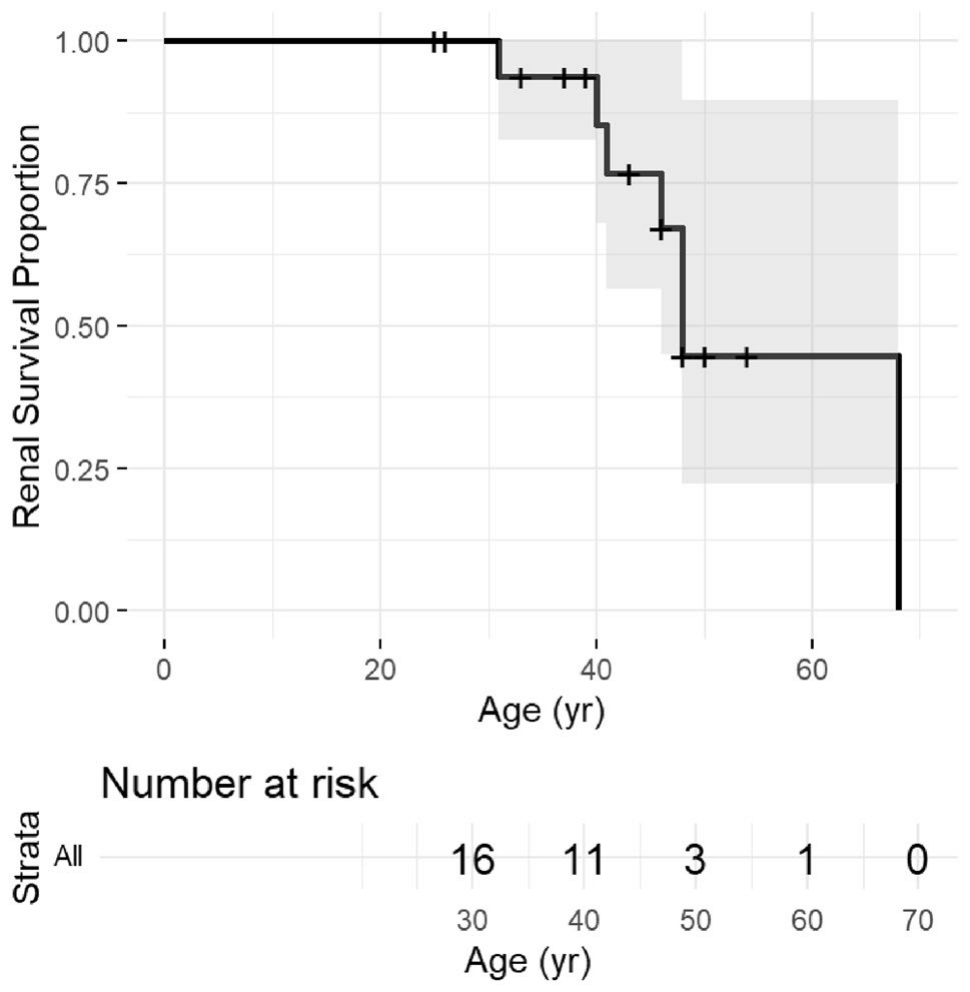
Kaplan–Meier survival curve. Kaplan–Meier survival curve illustrating the renal survival proportion as a function of patient age (years). The solid black line represents the estimated renal survival probability, with censored data points indicated by vertical tick marks. The shaded area denotes the 95% confidence interval (CI). The number at risk table below the plot indicates the number of patients still at risk at each corresponding time point. The median renal survival age, defined as the age at which 50% of patients develop ESKD, was 48 years (95% CI 46–NA years). The upper confidence interval could not be determined owing to limited event occurrences beyond this time point. As the follow-up period progresses, the number at risk decreases, leading to wider confidence intervals and reduced reliability of survival estimates at later ages

The clinical characteristics of the 19 cases diagnosed with long leads are listed in Table 1. The median age at diagnosis was 41 years (IQR: 35–48), with a male-to-female ratio of 11:8. A family history of CKD was present in 17 cases. Kidney biopsy was performed in 12 cases, of which 8 exhibited characteristic findings of medullary cystic kidney disease, including interstitial fibrosis and tubular atrophy. Hypertension was observed in 10 cases, and hyperuricemia was noted in 6 cases. At the time of diagnosis, renal replacement therapy had already been initiated in 7 cases. Among the 12 patients who had not undergone renal replacement therapy, only 5 exhibited proteinuria. The median age at the onset of ESKD was 48 years (95% CI: 46–NA years) in patients with ADTKD-*MUC1* (Fig. 3).

### Second approach (Fig. 2)

VNtyper-Kestrel was used to analyze short-read sequencing data for 976 cases, including 505 cases of suspected hereditary nephrotic syndrome, 313 cases of suspected hereditary Bartter syndrome, 115 cases of suspected Dent disease, and 43 cases of suspected hereditary renal tubular acidosis. Among these, VNtyper-Kestrel yielded positive results in 5 cases with suspected hereditary nephrotic syndrome, 2 cases with suspected hereditary Bartter syndrome, and 3 cases with suspected Dent disease. All VNtyper-positive cases were classified as low-confidence positives. The median coverages in the VNTR region of *MUC1* were 363 in cases of suspected hereditary nephrotic syndrome, 340 in cases of suspected Bartter syndrome, 318 in cases of suspected Dent disease, and 304 in cases of suspected hereditary renal tubular acidosis. However, no true *MUC1* pathogenic variants were identified using long-read sequencing. These findings suggest that all VNtyper-positive results for this cohort were false positives (Supplementary Table S6, S7, S8, S9).

## Discussion

In this study, we evaluated the performance of the VNtyper-Kestrel pipeline for the detection of pathogenic variants in the *MUC1* gene in clinical settings. Upon reviewing cases that tested positive using long-read sequencing, we identified instances where the VNtyper-Kestrel pipeline yielded negative results. In these cases, coverage was insufficient, and subsequent analyses using a newly developed ADTKD panel achieved adequate coverage, leading to positive results using the VNtyper-Kestrel pipeline. These finding suggests that the VNtyper-Kestrel pipeline can accurately identify true-positive cases when coverage is sufficient.

VNtyper-Kestrel is a novel computational approach for identifying *MUC1* variants using short-read sequencing data, as first described by Saei et al. in 2023 [18]. This method enables the detection of *MUC1* variants within highly repetitive tandem repeat sequences, which are traditionally challenging to analyze using PCR or mapping-based approaches. By circumventing the need for long-read sequencing technologies, VNtyper-Kestrel provides a cost-effective and clinically feasible alternative for *MUC1* variant detection. Its ability to reliably identify pathogenic variants without complex sequencing methodologies underscores its potential utility in routine clinical diagnostics.

We applied the VNtyper-Kestrel pipeline to screen cases that were not clinically diagnosed with ADTKD. Although some cases were flagged as positive using the VNtyper-Kestrel pipeline, all were categorized as low-confidence positives and were ultimately determined to be negative upon validation through long-read sequencing. Therefore, the potential for false-positive results must be considered when using the VNtyper-Kestrel pipeline. Interestingly, all false-positive variants identified in our study shared a common insertion, “GGCT,” and exhibited markedly low depth scores. Previous studies employing VNtyper-Kestrel have reported only single-base insertions or duplications, whereas large insertions such as “GGCT” have not been consistently observed. This suggests that such variants may represent artifacts generated by the pipeline. Incorporating filtering strategies that exclude variants with large insertions and low depth scores may help reduce the risk of false positives in future applications of VNtyper-Kestrel.

In a recent study [19], *MUC1* variants in 40 patients were identified using VNtyper-Kestrel, including variants in 33 new index patients and 7 relatives. Among the 33 index cases, ADTKD was suspected in 20 cases based on clinical features, while ADTKD was not considered in the remaining 13 cases. The detection rate among patients without a clinical suspicion of ADTKD varied by disease category, with rates of 0.05% in glomerular disease, 1.2% in ciliopathy, 0.09% in CAKUT, and 2.5% in CKD of unknown origin. Furthermore, six patients had no family history of kidney disease, and de novo presentation was confirmed in two patients through segregation studies. Given these findings, the lack of detection in patients without suspected ADTKD in our cohort is reasonable. Our cohort primarily consisted of pediatric patients with hereditary kidney diseases. It is possible that the detection rate of *MUC1* variants would be higher in adult patients with unexplained ESKD. Future studies focusing on such patient populations may provide further insights into the clinical utility of the VNtyper-Kestrel pipeline for detecting *MUC1* variants.

*MUC1* variants lead to the production and accumulation of aberrant proteins, which exert toxic effects on renal tubules. Given this pathogenic mechanism, the disease may have a long latency period before clinical symptoms appear. Recent advancements in therapeutic strategies have been reported. For example, Dvela-Levitt et al. [14] have reported promising small-molecule interventions targeting the intracellular accumulation of misfolded MUC1-fs proteins. These compounds, such as BRD4780, facilitate the lysosomal degradation of toxic proteins, potentially reversing disease progression. Screening asymptomatic individuals for *MUC1* variants using VNtyper-Kestrel and initiating early therapeutic interventions could reduce the number of patients progressing to ESKD. Therefore, VNtyper-based screening is expected to play an increasingly critical role in the management of ADTKD-*MUC1*.

This study had several limitations. First, VNtyper-Kestrel yielded false-negative results when the sequencing coverage was insufficient. In our analysis, there was variation in coverage among cases. Ensuring adequate and uniform coverage is crucial for minimizing false-negative results in future applications. It should be noted, though, that sufficient coverage will increase false positives. Second, our screening included cases in which pathogenic variants in other genes had already been identified. This approach may have resulted in unnecessary screening, potentially reducing the overall efficiency of the pipeline. Future studies should focus on refining the selection criteria for screening to enhance cost-effectiveness and clinical applicability. Third, only cases diagnosed with ADTKD-*MUC1* that underwent both short-read and long-read sequencing were included in this study. This approach excluded some cases, such as family members who underwent only one type of sequencing. Fourth, VNtyper-Kestrel utilizes known motif sequences as references, and thus is inherently limited in its ability to detect novel or previously uncharacterized VNTR motifs. As a result, population-specific or rare variants, particularly those unique to the Japanese population, may not be captured. Future methodological enhancements incorporating de novo motif discovery approaches would be beneficial to address this limitation.

In conclusion, the VNtyper-Kestrel pipeline offers a promising approach for detecting pathogenic *MUC1* variants in ADTKD using short-read sequencing. The pipeline demonstrated high sensitivity when sequencing coverage was adequate, highlighting its potential as a cost-effective screening tool. However, false-negative results in cases with insufficient coverage and false-positive results in non-ADTKD cohorts underscore the importance of confirmatory long-read sequencing in uncertain cases. Refining sequencing strategies and patient selection criteria will be crucial for improving the clinical applicability. Future research should focus on optimizing diagnostic accuracy and expanding its use to broader patient populations, particularly adults with unexplained kidney disease. By enhancing early detection, VNtyper-Kestrel could contribute to improved disease management and therapeutic interventions for ADTKD-*MUC1*.

## Supplementary Information

Below is the link to the electronic supplementary material.

Supplementary file1 (XLSX 82 KB)

Supplementary file2 (DOCX 45 KB)
