# Supplementary material for: Clinical use of the VNtyper-Kestrel pipeline for MUC1 variant detection in autosomal-dominant tubulointerstitial kidney disease
Source: Clin Exp Nephrol. 2025 Apr 17;29(9):1192–9. doi: 10.1007/s10157-025-02675-y (PMC12441061; doi:10.1007/s10157-025-02675-y)
Supplement: Supplementary file 2 — Supplementary file2 (DOCX 45 KB) [file 10157_2025_2675_MOESM2_ESM.docx]

Supplementary materials

**Clinical use of the VNtyper-Kestrel pipeline for *MUC1* variant detection in autosomal dominant tubulointerstitial kidney disease**

Contents

Supplementary Table S1A. The gene list constructed using HaloPlex (version 2, 128 genes)

Supplementary Table S1B. The gene list constructed using HaloPlex (version 4, 172 genes)

Supplementary Table S1C. The gene list constructed using HaloPlex (version 5, 159 genes)

Supplementary Table S1D. The gene list constructed using HaloPlex (version 6, 164 genes)

Supplementary Table S1E. The gene list constructed using HaloPlex (version 7, 181 genes)

Supplementary Table S1F. The gene list constructed using HaloPlex (version 8, 183 genes)

Supplementary Table S1G. The gene list constructed using SureSelect (version 9,　193genes)

Supplementary Table S1H. The gene list constructed using SureSelect (version 10, 188genes)

Supplementary Table S1I. The gene list constructed using SureSelect (version 11, 186genes)

Supplementary Table S1J. The gene list constructed using SureSelect (version 12, 187genes)

Supplementary Table S2. The gene list used in an ADTKD-specific panel

Supplementary Table S3. The gene list used in the second approach

Supplementary Table S4. Results of Long-Read Sequencing and VNtyper Analysis in Patients with Suspected ADTKD

Supplementary Table S5. Results of VNtyper Analysis using the ADTKD panel

Supplementary Table S6. Results of VNtyper Analysis in Patients with Suspected hereditary nephrotic syndrome

Supplementary Table S7. Results of VNtyper Analysis in Patients with Suspected Bartter syndrome

Supplementary Table S8. Results of VNtyper Analysis in Patients with Suspected Dent Disease

Supplementary Table S9. Results of VNtyper Analysis in Patients with Suspected hereditary renal tubular acidosis

Supplementary Table S1A. The gene list constructed using HaloPlex (version 2, 128 genes)

| *ACE* | *BMP7* | *CXCL12* | *FZD4* | *IQCB1* | *NPHS2* | *RET* | *TBX18* | *WNT4* |
| --- | --- | --- | --- | --- | --- | --- | --- | --- |
| *ACTN4* | *CC2D2A* | *CXCR4* | *FZD8* | *ITGA8* | *NUP107* | *ROBO2* | *TMEM138* | *WNT5A* |
| *ADCK4* | *CD2AP* | *DCDC2* | *GATA3* | *KAL1* | *NUP133* | *ROR1* | *TMEM231* | *WNT7A* |
| *AGT* | *CDC5L* | *DSTYK* | *GDNF* | *KIAA0586* | *NXF5* | *ROR2* | *TMEM237* | *WNT7B* |
| *AGTR1* | *CEP164* | *EP300* | *GLIS2* | *LAMB2* | *OFD1* | *RPGLIP1L* | *TMEM67* | *WNT9B* |
| *AGTR2* | *CEP290* | *EYA1* | *GREM1* | *LMNA* | *OSR1* | *SALL1* | *TNXB* | *WT1* |
| *AHI1* | *CEP41* | *FAT3* | *GRIP1* | *LMX1B* | *PAX2* | *SDCCAG8* | *TP53* | *ZEB2* |
| *ALG13* | *CEP83* | *FAT4* | *HNF1B* | *MDM2* | *PAX8* | *SIX1* | *TRAP1* | *ZNF423* |
| *ANKS3* | *CHD1L* | *FGF2* | *HOXA13* | *MKKS* | *PDE6D* | *SIX2* | *TRPC6* |  |
| *ANKS6* | *CHD4* | *FGF20* | *HOXD11* | *MUC1* | *PKD1* | *SIX5* | *TTC21B* |  |
| *ANLN* | *CHRM3* | *FGF9* | *IFN2* | *MYO1E* | *PKD2* | *SLIT2* | *UMOD* |  |
| *APOL1* | *CITED1* | *FOXD1* | *IFT172* | *NEK8* | *PKHD1* | *SOX17* | *UPK3A* |  |
| *ARL13B* | *COQ6* | *FRAS1* | *IFT81* | *NPHP1* | *PLCE1* | *SPRY1* | *VANGL2* |  |
| *BMP2* | *CSPP1* | *FREM1* | *INPP5E* | *NPHP3* | *PODXL* | *SRGAP1* | *WDR19* |  |
| *BMP4* | *CTDNEP1* | *FREM2* | *INVS* | *NPHP4* | *REN* | *TBX1* | *WNT11* |  |

Supplementary Table S1B. The gene list constructed using HaloPlex (version 4, 172 genes)

| *ACE* | *B9D1* | *CD2AP* | *CTDNEP1* | *FOXD1* | *IFN2* | *LMNA* | *NUP107* | *RET* | *TBX1* | *TSC1* | *WNT7B* |
| --- | --- | --- | --- | --- | --- | --- | --- | --- | --- | --- | --- |
| *ACTN4* | *B9D2* | *CDC5L* | *CXCL12* | *FRAS1* | *IFT27* | *LMX1B* | *NUP133* | *ROBO2* | *TBX18* | *TSC2* | *WNT9B* |
| *ADCK4* | *BBIP1* | *CENPF* | *CXCR4* | *FREM1* | *IFT43* | *LZTFL1* | *NXF5* | *ROR1* | *TCTN2* | *TTC8* | *WNT11* |
| *AGT* | *BBS1* | *CEP41* | *DCDC2* | *FREM2* | *IFT81* | *MDM2* | *OFD1* | *ROR2* | *TCTN3* | *TTC21B* | *WT1* |
| *AGTR1* | *BBS2* | *CEP83* | *DDX59* | *FZD4* | *IFT122* | *MKKS* | *OSR1* | *RPGLIP1L* | *TMEM67* | *UMOD* | *XPNPEP3* |
| *AGTR2* | *BBS4* | *CEP104* | *DSTYK* | *FZD8* | *IFT140* | *MKS1* | *PAX2* | *SALL1* | *TMEM138* | *UPK3A* | *ZEB2* |
| *AHI1* | *BBS5* | *CEP120* | *DYNC2H1* | *GANAB* | *IFT172* | *MUC1* | *PAX8* | *SDCCAG8* | *TMEM216* | *VANGL2* | *ZNF423* |
| *ALG13* | *BBS7* | *CEP164* | *EP300* | *GATA3* | *INPP5E* | *MYO1E* | *PDE6D* | *SIX1* | *TMEM231* | *WDPCP* |  |
| *ALMS1* | *BBS10* | *CEP290* | *EYA1* | *GDNF* | *INVS* | *NEK1* | *PKD1* | *SIX2* | *TMEM237* | *WDR19* |  |
| *ANKS3* | *BBS12* | *CHD1L* | *FAT3* | *GLIS2* | *IQCB1* | *NEK8* | *PKD2* | *SIX5* | *TNXB* | *WDR34* |  |
| *ANKS6* | *BMP2* | *CHD4* | *FAT4* | *GREM1* | *ITGA8* | *NODAL* | *PKHD1* | *SLIT2* | *TP53* | *WDR35* |  |
| *ANLN* | *BMP4* | *CHRM3* | *FGF2* | *GRIP1* | *KAL1* | *NPHP1* | *PLCE1* | *SOX17* | *TRAF3IP1* | *WDR60* |  |
| *APOL1* | *BMP7* | *CITED1* | *FGF9* | *HOXA13* | *KIAA0586* | *NPHP3* | *PODXL* | *SPRY1* | *TRAP1* | *WNT4* |  |
| *ARL6* | *C5orf42* | *COQ6* | *FGF20* | *HOXD11* | *KIF14* | *NPHP4* | *PTHB1* | *SRGAP1* | *TRIM32* | *WNT5A* |  |
| *ARL13B* | *CC2D2A* | *CSPP1* | *FGFR2* | *HNF1B* | *LAMB2* | *NPHS2* | *REN* | *TBC1D1* | *TRPC6* | *WNT7A* |  |

Supplementary Table S1C. The gene list constructed using HaloPlex (version 5, 159 genes)

| *ACE* | *BBS1* | *CEP41* | *DYNC2H1* | *GANAB* | *IFT80* | *LMNA* | *PDE6D* | *SIX1* | *TMEM107* | *WDPCP* |
| --- | --- | --- | --- | --- | --- | --- | --- | --- | --- | --- |
| *AGT* | *BBS2* | *CEP83* | *DZIP1L* | *GATA3* | *IFT81* | *LMX1B* | *PIBF1* | *SIX2* | *TMEM138* | *WDR19* |
| *AGTR1* | *BBS4* | *CEP104* | *EP300* | *GDNF* | *IFT122* | *LZTFL1* | *PKD1* | *SIX5* | *TMEM216* | *WDR34* |
| *AGTR2* | *BBS5* | *CEP120* | *EVC* | *GLIS2* | *IFT140* | *MKKS* | *PKD2* | *SLIT2* | *TMEM231* | *WDR35* |
| *AHI1* | *BBS7* | *CEP164* | *EVC2* | *GLIS3* | *IFT172* | *MKS1* | *PKHD1* | *SOX17* | *TMEM237* | *WDR60* |
| *ALG9* | *BBS10* | *CEP290* | *EXOC4* | *GRIP1* | *INPP5E* | *MUC1* | *PTHB1* | *SPRY1* | *TNXB* | *WNT4* |
| *ALMS1* | *BBS12* | *CHD1L* | *EXOC8* | *GRLF1* | *INVS* | *NEK1* | *REN* | *SRGAP1* | *TRAF3IP1* | *WT1* |
| *ANKS3* | *C2CD3* | *CHD4* | *EYA1* | *HNF1B* | *IQCB1* | *NEK8* | *RET* | *TBC1D1* | *TRIM32* | *XPNPEP3* |
| *ANKS6* | *C5orf42* | *CHRM3* | *FAN1* | *HOXA13* | *ITGA8* | *NPHP1* | *ROBO2* | *TBC1D32* | *TSC1* | *ZNF423* |
| *ARL6* | *C21orf2* | *CITED1* | *FGF9* | *HPRT1* | *JAG1* | *NPHP3* | *RPGLIP1L* | *TBX1* | *TSC2* |  |
| *ARL13B* | *CC2D2A* | *CSPP1* | *FGF20* | *HYLS1* | *KAL1* | *NPHP4* | *SALL1* | *TBX18* | *TTBK2* |  |
| *ATXN10* | *CCDC28B* | *CTDNEP1* | *FGFR2* | *ICK* | *KIAA0586* | *OFD1* | *SARS2* | *TCTN1* | *TTC8* |  |
| *B9D1* | *CDC5L* | *DCDC2* | *FRAS1* | *IFN2* | *KIF7* | *PAX2* | *SCLT1* | *TCTN2* | *TTC21B* |  |
| *B9D2* | *CENPF* | *DDX59* | *FREM1* | *IFT27* | *KIF14* | *PAX8* | *SDCCAG8* | *TCTN3* | *UMOD* |  |
| *BBIP1* | *CEP19* | *DSTYK* | *FREM2* | *IFT43* | *LIFR* | *PBX1* | *SEC61A1* | *TMEM67* | *VANGL2* |  |

Supplementary Table S1D. The gene list constructed using HaloPlex (version 6, 164 genes)

| *ACE* | *BBS1* | *CEP41* | *DYNC2H1* | *GANAB* | *IFT80* | *LMNA* | *PDE6D* | *SIX1* | *TMEM107* | *WDPCP* |
| --- | --- | --- | --- | --- | --- | --- | --- | --- | --- | --- |
| *AGT* | *BBS2* | *CEP83* | *DZIP1L* | *GATA3* | *IFT81* | *LMX1B* | *PIBF1* | *SIX2* | *TMEM138* | *WDR19* |
| *AGTR1* | *BBS4* | *CEP104* | *EP300* | *GDNF* | *IFT122* | *LZTFL1* | *PKD1* | *SIX5* | *TMEM216* | *WDR34* |
| *AGTR2* | *BBS5* | *CEP120* | *EVC* | *GLIS2* | *IFT140* | *MKKS* | *PKD2* | *SLIT2* | *TMEM231* | *WDR35* |
| *AHI1* | *BBS7* | *CEP164* | *EVC2* | *GLIS3* | *IFT172* | *MKS1* | *PKHD1* | *SOX17* | *TMEM237* | *WDR60* |
| *ALG9* | *BBS10* | *CEP290* | *EXOC4* | *GRIP1* | *INPP5E* | *MUC1* | *PTHB1* | *SPRY1* | *TNXB* | *WNT4* |
| *ALMS1* | *BBS12* | *CHD1L* | *EXOC8* | *GRLF1* | *INVS* | *NEK1* | *REN* | *SRGAP1* | *TRAF3IP1* | *WT1* |
| *ANKS3* | *C2CD3* | *CHD4* | *EYA1* | *HNF1B* | *IQCB1* | *NEK8* | *RET* | *TBC1D1* | *TRIM32* | *XPNPEP3* |
| *ANKS6* | *C5orf42* | *CHRM3* | *FAN1* | *HOXA13* | *ITGA8* | *NPHP1* | *ROBO2* | *TBC1D32* | *TSC1* | *ZNF423* |
| *ARL6* | *C21orf2* | *CITED1* | *FGF9* | *HPRT1* | *JAG1* | *NPHP3* | *RPGLIP1L* | *TBX1* | *TSC2* |  |
| *ARL13B* | *CC2D2A* | *CSPP1* | *FGF20* | *HYLS1* | *KAL1* | *NPHP4* | *SALL1* | *TBX18* | *TTBK2* |  |
| *ATXN10* | *CCDC28B* | *CTDNEP1* | *FGFR2* | *ICK* | *KIAA0586* | *OFD1* | *SARS2* | *TCTN1* | *TTC8* |  |
| *B9D1* | *CDC5L* | *DCDC2* | *FRAS1* | *IFN2* | *KIF7* | *PAX2* | *SCLT1* | *TCTN2* | *TTC21B* |  |
| *B9D2* | *CENPF* | *DDX59* | *FREM1* | *IFT27* | *KIF14* | *PAX8* | *SDCCAG8* | *TCTN3* | *UMOD* |  |
| *BBIP1* | *CEP19* | *DSTYK* | *FREM2* | *IFT43* | *LIFR* | *PBX1* | *SEC61A1* | *TMEM67* | *VANGL2* |  |

Supplementary Table S1E. The gene list constructed using HaloPlex (version 7, 181 genes)

| *ACE* | *BBS1* | *CEP83* | *EP300* | *GFRA1* | *IFT80* | *LRIG2* | *PIBF1* | *SOX11* | *TNXB* |
| --- | --- | --- | --- | --- | --- | --- | --- | --- | --- |
| *ACTG2* | *BBS2* | *CEP104* | *EVC* | *GLIS2* | *IFT81* | *LRP5* | *PKD1* | *SOX17* | *TRAF3IP1* |
| *AGT* | *BBS4* | *CEP120* | *EVC2* | *GLIS3* | *IFT122* | *LZTFL1* | *PKD2* | *SPRY1* | *TRIM32* |
| *AGTR1* | *BBS5* | *CEP164* | *EXOC4* | *GPC3* | *IFT140* | *MAPKBP1* | *PKHD1* | *SRGAP1* | *TSC1* |
| *AGTR2* | *BBS7* | *CEP290* | *EXOC8* | *GREB1L* | *IFT172* | *MKKS* | *REN* | *SUFU* | *TSC2* |
| *AHI1* | *BBS9* | *CHD1L* | *EYA1* | *GREM1* | *INPP5E* | *MKS1* | *RET* | *TBC1D32* | *TTC8* |
| *ALG8* | *BBS10* | *CHD4* | *FAN1* | *GRIP1* | *INTU* | *MUC1* | *ROBO2* | *TBX1* | *TTC21B* |
| *ALG9* | *BBS12* | *CHD7* | *FGF20* | *HNF1B* | *INVS* | *NEK1* | *RPGRIP1L* | *TBX18* | *UMOD* |
| *ALMS1* | *BICC1* | *CHRM3* | *FGFR1* | *HOXA13* | *IQCB1* | *NEK8* | *SALL1* | *TCTEX1D2* | *UPK3A* |
| *ANKS6* | *C2CD3* | *CITED1* | *FGFR2* | *HPRT1* | *ITGA8* | *NOTCH2* | *SARS2* | *TCTN1* | *VANGL2* |
| *ARL3* | *C5orf42* | *CRB2* | *FRAS1* | *HPSE2* | *JAG1* | *NPHP1* | *SCLT1* | *TCTN2* | *WDPCP* |
| *ARL6* | *C8orf37* | *CSPP1* | *FREM1* | *HYLS1* | *KAL1* | *NPHP3* | *SDCCAG8* | *TCTN3* | *WDR19* |
| *ARL13B* | *CC2D2A* | *DCDC2* | *FREM2* | *INF2* | *KIAA0556* | *NPHP4* | *SEC61A1* | *TMEM67* | *WDR34* |
| *ARMC9* | *CCDC28B* | *DDX59* | *GANAB* | *IFT27* | *KIAA0586* | *OFD1* | *SIX1* | *TMEM107* | *WDR35* |
| *ATXN10* | *CDC5L* | *DNAJB11* | *GATA3* | *IFT43* | *KIAA0753* | *PAX2* | *SIX2* | *TMEM138* | *WDR60* |
| *B9D1* | *CDKN1C* | *DSTYK* | *GDF11* | *IFT52* | *KIF7* | *PAX8* | *SIX5* | *TMEM216* | *WNT4* |
| *B9D2* | *CENPF* | *DYNC2H1* | *GDNF* | *IFT57* | *KIF14* | *PBX1* | *SLIT2* | *TMEM231* | *WT1* |
| *BBIP1* | *CEP41* | *DZIP1L* | *GEN1* | *IFT74* | *LMX1B* | *PDE6D* | *SOX9* | *TMEM237* | *XPNPEP3* |
|  |  |  |  |  |  |  |  |  | *ZNF423* |

Supplementary Table S1F. The gene list constructed using HaloPlex (version 8, 183 genes)

| *ACE* | *ATXN10* | *C8orf37* | *CHRM3* | *EYA1* | *GLIS3* | *IFT74* | *KIAA0753* | *NPHP3* | *SALL1* | *SUFU* | *TRAF3IP1* | *WT1* |
| --- | --- | --- | --- | --- | --- | --- | --- | --- | --- | --- | --- | --- |
| *ACTG2* | *B9D1* | *CC2D2A* | *CITED1* | *FAN1* | *GPC3* | *IFT80* | *KIF7* | *NPHP4* | *SARS2* | *TBC1D32* | *TRIM32* | *XPNPEP3* |
| *ADAMTS9* | *B9D2* | *CCDC28B* | *CRB2* | *FGF20* | *GREB1L* | *IFT81* | *KIF14* | *OFD1* | *SCLT1* | *TBX1* | *TSC1* | *ZNF423* |
| *AGT* | *BBIP1* | *CDC5L* | *CSPP1* | *FGFR1* | *GREM1* | *IFT122* | *LMX1B* | *PAX2* | *SDCCAG8* | *TBX18* | *TSC2* |  |
| *AGTR1* | *BBS1* | *CDKN1C* | *DCDC2* | *FGFR2* | *GRIP1* | *IFT140* | *LRIG2* | *PAX8* | *SEC61A1* | *TCTEX1D2* | *TTC8* |  |
| *AGTR2* | *BBS2* | *CENPF* | *DDX59* | *FRAS1* | *HNF1B* | *IFT172* | *LRP5* | *PBX1* | *SIX1* | *TCTN1* | *TTC21B* |  |
| *AHI1* | *BBS4* | *CEP41* | *DNAJB11* | *FREM1* | *HOXA13* | *INPP5E* | *LZTFL1* | *PDE6D* | *SIX2* | *TCTN2* | *UMOD* |  |
| *ALG8* | *BBS5* | *CEP83* | *DSTYK* | *FREM2* | *HPRT1* | *INTU* | *MAPKBP1* | *PIBF1* | *SIX5* | *TCTN3* | *UPK3A* |  |
| *ALG9* | *BBS7* | *CEP104* | *DYNC2H1* | *GANAB* | *HPSE2* | *INVS* | *MKKS* | *PKD1* | *SLIT2* | *TMEM67* | *VANGL2* |  |
| *ALMS1* | *BBS9* | *CEP120* | *DZIP1L* | *GATA3* | *HYLS1* | *IQCB1* | *MKS1* | *PKD2* | *SON* | *TMEM107* | *WDPCP* |  |
| *ANKS6* | *BBS10* | *CEP164* | *EP300* | *GDF11* | *INF2* | *ITGA8* | *MUC1* | *PKHD1* | *SOX9* | *TMEM138* | *WDR19* |  |
| *ARL3* | *BBS12* | *CEP290* | *EVC* | *GDNF* | *IFT27* | *JAG1* | *NEK1* | *REN* | *SOX11* | *TMEM216* | *WDR34* |  |
| *ARL6* | *BICC1* | *CHD1L* | *EVC2* | *GEN1* | *IFT43* | *KAL1* | *NEK8* | *RET* | *SOX17* | *TMEM231* | *WDR35* |  |
| *ARL13B* | *C2CD3* | *CHD4* | *EXOC4* | *GFRA1* | *IFT52* | *KIAA0556* | *NOTCH2* | *ROBO2* | *SPRY1* | *TMEM237* | *WDR60* |  |
| *ARMC9* | *C5orf42* | *CHD7* | *EXOC8* | *GLIS2* | *IFT57* | *KIAA0586* | *NPHP1* | *RPGRIP1L* | *SRGAP1* | *TNXB* | *WNT4* |  |

Supplementary Table S1G. The gene list constructed using HaloPlex (version 9, 193genes)

| *ACE* | *BBS2* | *CEP164* | *EVC2* | *GPC3* | *IFT172* | *MKS1* | *RET* | *TBC1D32* | *TTC21B* |
| --- | --- | --- | --- | --- | --- | --- | --- | --- | --- |
| *ACTG2* | *BBS4* | *CEP290* | *EXOC4* | *GREB1L* | *INPP5E* | *MUC1* | *ROBO2* | *TBX1* | *UMOD* |
| *ADAMTS9* | *BBS5* | *CHD1L* | *EXOC8* | *GREM1* | *INTU* | *NEK1* | *RPGRIP1L* | *TBX6* | *UPK3A* |
| *AGT* | *BBS7* | *CHD4* | *EYA1* | *GRIP1* | *INVS* | *NEK8* | *SALL1* | *TBX18* | *VANGL2* |
| *AGTR1* | *BBS9* | *CHD7* | *FAM149B1* | *HNF1B* | *IQCB1* | *NOTCH2* | *SARS2* | *TCTEX1D2* | *WDPCP* |
| *AGTR2* | *BBS10* | *CHRM3* | *FAN1* | *HOXA11* | *ITGA8* | *NPHP1* | *SCLT1* | *TCTN1* | *WDR19* |
| *AHI1* | *BBS12* | *CHRNA3* | *FGF20* | *HOXA13* | *JAG1* | *NPHP3* | *SDCCAG8* | *TCTN2* | *WDR34* |
| *ALG8* | *BICC1* | *CLCN5* | *FGFR1* | *HPRT1* | *KAL1* | *NPHP4* | *SEC61A1* | *TCTN3* | *WDR35* |
| *ALG9* | *C2CD3* | *CITED1* | *FGFR2* | *HPSE2* | *KIAA0556* | *NRIP1* | *SIX1* | *TMEM67* | *WDR60* |
| *ALMS1* | *C5orf42* | *CRB2* | *FRAS1* | *HYLS1* | *KIAA0586* | *OCRL* | *SIX2* | *TMEM107* | *WNT4* |
| *ANKS6* | *C8orf37* | *CSPP1* | *FREM1* | *INF2* | *KIAA0753* | *OFD1* | *SIX5* | *TMEM138* | *WT1* |
| *ARL3* | *CC2D2A* | *DCDC2* | *FREM2* | *IFT27* | *KIF7* | *PAX2* | *SLC20A1* | *TMEM216* | *XPNPEP3* |
| *ARL6* | *CCDC28B* | *DDX59* | *GANAB* | *IFT43* | *KIF14* | *PAX8* | *SLIT2* | *TMEM231* | *ZNF423* |
| *ARL13B* | *CDC5L* | *DNAJB11* | *GATA3* | *IFT52* | *LMX1B* | *PBX1* | *SON* | *TMEM237* |  |
| *ARMC9* | *CDKN1C* | *DSTYK* | *GDF11* | *IFT57* | *LRIG2* | *PDE6D* | *SOX9* | *TNXB* |  |
| *ATXN10* | *CENPF* | *DYNC2H1* | *GDNF* | *IFT74* | *LRP5* | *PIBF1* | *SOX11* | *TRAF3IP1* |  |
| *B9D1* | *CEP41* | *DYNC2LI1* | *GEN1* | *IFT80* | *LZTFL1* | *PKD1* | *SOX17* | *TRIM32* |  |
| *B9D2* | *CEP83* | *DZIP1L* | *GFRA1* | *IFT81* | *MAFB* | *PKD2* | *SPRY1* | *TSC1* |  |
| *BBIP1* | *CEP104* | *EP300* | *GLIS2* | *IFT122* | *MAPKBP1* | *PKHD1* | *SRGAP1* | *TSC2* |  |
| *BBS1* | *CEP120* | *EVC* | *GLIS3* | *IFT140* | *MKKS* | *REN* | *SUFU* | *TTC8* |  |

Supplementary Table S1H. The gene list constructed using SureSelect (version 10, 188genes)

| *ACE* | *B9D2* | *CDC5L* | *CRB2* | *FAN1* | *GRIP1* | *IFT140* | *LRIG2* | *NPHP4* | *SALL1* | *TCTN2* | *UMOD* |
| --- | --- | --- | --- | --- | --- | --- | --- | --- | --- | --- | --- |
| *ACTG2* | *BBIP1* | *CDKN1C* | *CSPP1* | *FGF20* | *HNF1B* | *IFT172* | *LRP5* | *NRIP1* | *SARS2* | *TCTN3* | *UPK3A* |
| *AGT* | *BBS1* | *CENPF* | *DCDC2* | *FGFR1* | *HOXA11* | *INPP5E* | *LZTFL1* | *OCRL* | *SCLT1* | *TMEM67* | *VANGL2* |
| *AGTR1* | *BBS2* | *CEP41* | *DDX59* | *FGFR2* | *HOXA13* | *INTU* | *MAFB* | *OFD1* | *SDCCAG8* | *TMEM107* | *WDPCP* |
| *AGTR2* | *BBS4* | *CEP83* | *DNAJB11* | *FRAS1* | *HPRT1* | *INVS* | *MAPKBP1* | *PAX2* | *SEC61A1* | *TMEM138* | *WDR19* |
| *AHI1* | *BBS5* | *CEP104* | *DSTYK* | *FREM1* | *HPSE2* | *IQCB1* | *MKKS* | *PAX8* | *SIX1* | *TMEM216* | *WDR34* |
| *ALG8* | *BBS7* | *CEP120* | *DYNC2H1* | *FREM2* | *HYLS1* | *ITGA8* | *MKS1* | *PBX1* | *SIX2* | *TMEM231* | *WDR35* |
| *ALG9* | *BBS9* | *CEP164* | *DYNC2LI1* | *GANAB* | *INF2* | *JAG1* | *MUC1* | *PDE6D* | *SIX5* | *TMEM237* | *WDR60* |
| *ALMS1* | *BBS10* | *CEP290* | *DZIP1L* | *GATA3* | *IFT27* | *KAL1* | *MYH11* | *PIBF1* | *SON* | *TNXB* | *WNT4* |
| *ANKS6* | *BBS12* | *CFAP418* | *EP300* | *GDF11* | *IFT43* | *KIAA0556* | *MYL9* | *PKD1* | *SOX9* | *TOGARAM1* | *WT1* |
| *ARL3* | *BICC1* | *CHD1L* | *EVC* | *GDNF* | *IFT52* | *KIAA0586* | *MYLK* | *PKD2* | *SOX17* | *TRAF3IP1* | *XPNPEP3* |
| *ARL6* | *C2CD3* | *CHD4* | *EVC2* | *GFRA1* | *IFT57* | *KIAA0753* | *NEK1* | *PKHD1* | *SUFU* | *TRIM32* | *ZNF423* |
| *ARL13B* | *C5orf42* | *CHD7* | *EXOC4* | *GLIS2* | *IFT74* | *KIF7* | *NEK8* | *REN* | *TBX1* | *TSC1* |  |
| *ARMC9* | *CBWD1* | *CHRM3* | *EXOC8* | *GLIS3* | *IFT80* | *KIF14* | *NOTCH2* | *RET* | *TBX18* | *TSC2* |  |
| *ATXN10* | *CC2D2A* | *CHRNA3* | *EYA1* | *GPC3* | *IFT81* | *LMOD1* | *NPHP1* | *ROBO2* | *TCTEX1D2* | *TTC8* |  |
| *B9D1* | *CCDC28B* | *CLCN5* | *FAM149B1* | *GREB1L* | *IFT122* | *LMX1B* | *NPHP3* | *RPGRIP1L* | *TCTN1* | *TTC21B* |  |

Supplementary Table S1I. The gene list constructed using SureSelect (version 11, 186genes)

| *ACE* | *BBIP1* | *CENPF* | *DDX59* | *FGFR2* | *HOXA13* | *INTU* | *MAFB* | *OFD1* | *SDCCAG8* | *TMEM138* | *VANGL2* |
| --- | --- | --- | --- | --- | --- | --- | --- | --- | --- | --- | --- |
| *ACTG2* | *BBS1* | *CEP41* | *DNAJB11* | *FRAS1* | *HPRT1* | *INVS* | *MAPKBP1* | *PAX2* | *SEC61A1* | *TMEM216* | *WDPCP* |
| *AGT* | *BBS2* | *CEP83* | *DSTYK* | *FREM1* | *HPSE2* | *IQCB1* | *MKKS* | *PAX8* | *SIX1* | *TMEM218* | *WDR19* |
| *AGTR1* | *BBS4* | *CEP104* | *DYNC2H1* | *FREM2* | *HYLS1* | *ITGA8* | *MKS1* | *PBX1* | *SIX2* | *TMEM231* | *WDR34* |
| *AGTR2* | *BBS5* | *CEP120* | *DYNC2LI1* | *GANAB* | *INF2* | *JAG1* | *MUC1* | *PDE6D* | *SIX5* | *TMEM237* | *WDR35* |
| *AHI1* | *BBS7* | *CEP164* | *DZIP1L* | *GATA3* | *IFT27* | *KAL1* | *MYH11* | *PIBF1* | *SOX9* | *TNXB* | *WDR60* |
| *ALG8* | *BBS9* | *CEP290* | *EP300* | *GDF11* | *IFT43* | *KIAA0556* | *MYL9* | *PKD1* | *SOX17* | *TOGARAM1* | *WNT4* |
| *ALG9* | *BBS10* | *CFAP418* | *EVC* | *GDNF* | *IFT52* | *KIAA0586* | *MYLK* | *PKD2* | *SUFU* | *TRAF3IP1* | *WT1* |
| *ALMS1* | *BBS12* | *CHD4* | *EVC2* | *GFRA1* | *IFT57* | *KIAA0753* | *NEK1* | *PKHD1* | *TBX1* | *TRIM32* | *XPNPEP3* |
| *ANKS6* | *BICC1* | *CHD7* | *EXOC4* | *GLIS2* | *IFT74* | *KIF7* | *NEK8* | *REN* | *TBX18* | *TSC1* | *ZNF423* |
| *ARL3* | *C2CD3* | *CHRM3* | *EXOC8* | *GLIS3* | *IFT80* | *KIF14* | *NOTCH2* | *RET* | *TCTEX1D2* | *TSC2* |  |
| *ARL6* | *C5orf42* | *CHRNA3* | *EYA1* | *GPC3* | *IFT81* | *LMOD1* | *NPHP1* | *ROBO2* | *TCTN1* | *TTC8* |  |
| *ARL13B* | *CBWD1* | *CLCN5* | *FAM149B1* | *GREB1L* | *IFT122* | *LMX1B* | *NPHP3* | *RPGRIP1L* | *TCTN2* | *TTC21B* |  |
| *ARMC9* | *CC2D2A* | *CRB2* | *FAN1* | *GRIP1* | *IFT140* | *LRIG2* | *NPHP4* | *SALL1* | *TCTN3* | *TXNDC15* |  |
| *B9D1* | *CCDC28B* | *CSPP1* | *FGF20* | *HNF1B* | *IFT172* | *LRP5* | *NRIP1* | *SARS2* | *TMEM67* | *UMOD* |  |
| *B9D2* | *CDKN1C* | *DCDC2* | *FGFR1* | *HOXA11* | *INPP5E* | *LZTFL1* | *OCRL* | *SCLT1* | *TMEM107* | *UPK3A* |  |

Supplementary Table S1J. The gene list constructed using SureSelect (version 12, 187genes)

| *ACE* | *BBIP1* | *CEP41* | *DYNC2H1* | *GATA3* | *IFT57* | *LMOD1* | *NPHP4* | *SARS2* | *TMEM216* | *WDR34* |
| --- | --- | --- | --- | --- | --- | --- | --- | --- | --- | --- |
| *ACTG2* | *BBS1* | *CEP83* | *DYNC2LI1* | *GDF11* | *IFT74* | *LMX1B* | *NRIP1* | *SCLT1* | *TMEM218* | *WDR35* |
| *AGT* | *BBS2* | *CEP104* | *DZIP1L* | *GDNF* | *IFT80* | *LRIG2* | *OCRL* | *SDCCAG8* | *TMEM231* | *WDR60* |
| *AGTR1* | *BBS4* | *CEP120* | *EP300* | *GFRA1* | *IFT81* | *LRP5* | *OFD1* | *SEC61A1* | *TMEM237* | *WNT4* |
| *AGTR2* | *BBS5* | *CEP164* | *EVC* | *GLIS2* | *IFT122* | *LZTFL1* | *PAX2* | *SIX1* | *TNXB* | *WT1* |
| *AHI1* | *BBS7* | *CEP290* | *EVC2* | *GPC3* | *IFT140* | *MAFB* | *PAX8* | *SIX2* | *TOGARAM1* | *XPNPEP3* |
| *ALG5* | *BBS9* | *CHD4* | *EXOC4* | *GREB1L* | *IFT172* | *MAPKBP1* | *PBX1* | *SIX5* | *TRAF3IP1* | *ZNF423* |
| *ALG8* | *BBS10* | *CHD7* | *EXOC8* | *GRIP1* | *INPP5E* | *MKKS* | *PDE6D* | *SOX17* | *TRIM32* |  |
| *ALG9* | *BBS12* | *CHRM3* | *EYA1* | *HNF1B* | *INTU* | *MKS1* | *PIBF1* | *SUFU* | *TSC1* |  |
| *ALMS1* | *BICC1* | *CHRNA3* | *FAM149B1* | *HOXA11* | *INVS* | *MUC1* | *PKD1* | *TBX1* | *TSC2* |  |
| *ANKS6* | *C2CD3* | *CLCN5* | *FAN1* | *HOXA13* | *IQCB1* | *MYH11* | *PKD2* | *TBX18* | *TTC8* |  |
| *ANOS1* | *C5orf42* | *CRB2* | *FGF20* | *HPRT1* | *ITGA8* | *MYL9* | *PKHD1* | *TCTEX1D2* | *TTC21B* |  |
| *ARL3* | *C8orf37* | *CSPP1* | *FGFR1* | *HPSE2* | *JAG1* | *MYLK* | *REN* | *TCTN1* | *TXNDC15* |  |
| *ARL6* | *CBWD1* | *DACT1* | *FGFR2* | *HYLS1* | *KIAA0556* | *NEK1* | *RET* | *TCTN2* | *UMOD* |  |
| *ARL13B* | *CC2D2A* | *DCDC2* | *FRAS1* | *INF2* | *KIAA0586* | *NEK8* | *ROBO1* | *TCTN3* | *UPK3A* |  |
| *ARMC9* | *CCDC28B* | *DDX59* | *FREM1* | *IFT27* | *KIAA0753* | *NOTCH2* | *ROBO2* | *TMEM67* | *VANGL2* |  |
| *B9D1* | *CDKN1C* | *DNAJB11* | *FREM2* | *IFT43* | *KIF7* | *NPHP1* | *RPGRIP1L* | *TMEM107* | *WDPCP* |  |
| *B9D2* | *CENPF* | *DSTYK* | *GANAB* | *IFT52* | *KIF14* | *NPHP3* | *SALL1* | *TMEM138* | *WDR19* |  |

Supplementary Table S2. The gene list used in an ADTKD-specific panel

| *MUC1* | *UMOD* | *HNF1B* | *REN* | *SEC61A1* | *DNAJB11* |
| --- | --- | --- | --- | --- | --- |

Supplementary Table S3. The gene list used in the second approach

| *ACTN4* | *CD2AP* | *CTNS* | *HNF4A* | *MAGED2* | *PAX2* | *SLC34A1* |
| --- | --- | --- | --- | --- | --- | --- |
| *ADAMTS13* | *CDK20* | *CUBN* | *INF2* | *MAGI2* | *PCBD1* | *SLC4A1* |
| *ADCK4* | *CFB* | *CUL3* | *ITGA3* | *MCP* | *PDSS2* | *SLC4A4* |
| *ANK3* | *CFH* | *DAAM2* | *ITGB4* | *MUC1* | *PLCE1* | *SMARCAL1* |
| *ANKFY1* | *CFHR1* | *DGKE* | *ITSN1* | *MYH9* | *PODXL* | *TBC1D8B* |
| *ANLN* | *CFI* | *DLC1* | *ITSN2* | *MYO1E* | *PRDM15* | *THBD* |
| *AQP2* | *CFTR* | *EGF* | *KANK1* | *NDUFAF6* | *PTPRO* | *TNS2* |
| *ARHGAP24* | *CLCN5* | *EHD1* | *KANK2* | *NOS1AP* | *REN* | *TP53RK* |
| *ARHGDIA* | *CLCNKA* | *EHHADH* | *KANK4* | *NPHS1* | *RRAGD* | *TPRKB* |
| *ATP6V0A4* | *CLCNKB* | *EMP2* | *KCNA1* | *NPHS2* | *SCARB2* | *TRIM8* |
| *ATP6V1B1* | *CLDN10* | *EYA1* | *KCNJ1* | *NR3C2* | *SCNN1A* | *TRPC6* |
| *AVIL* | *CLDN16* | *FAT1* | *KCNJ10* | *NUP107* | *SCNN1B* | *TRPM6* |
| *AVP* | *CLDN19* | *FN1* | *KIRREL1* | *NUP133* | *SCNN1G* | *TTC21B* |
| *AVPR2* | *CNNM2* | *FXYD2* | *KLHL3* | *NUP160* | *SEC61A1* | *UMOD* |
| *BCS1L* | *COL4A3* | *GAPVD1* | *LAGE3* | *NUP205* | *SGPL1* | *WDR4* |
| *BSND* | *COL4A4* | *GATM* | *LAMA5* | *NUP85* | *SIX2* | *WDR73* |
| *C3* | *COL4A5* | *GLA* | *LAMB2* | *NUP93* | *SLC12A1* | *WNK1* |
| *CA2* | *COQ2* | *GLEPP1* | *LMNA* | *OCRL* | *SLC12A3* | *WNK4* |
| *CASR* | *COQ6* | *GON7* | *LMX1B* | *OSGEP* | *SLC26A3* | *WT1* |
| *CD151* | *CRB2* | *HNF1B* | *MAFB* | *P3H2* | *SLC2A2* | *XPO5* |
|  |  |  |  |  |  | *YRDC* |
